# *Amaranthus mangostanus* Inhibits the Differentiation of Osteoclasts and Prevents Ovariectomy-Induced Bone Loss

**DOI:** 10.1155/2020/1927017

**Published:** 2020-02-07

**Authors:** Yeon-Hui Jeong, Haeng Jeon Hur, Ae Sin Lee, Sang Hee Lee, Mi Jeong Sung

**Affiliations:** Research Group of Natural Materials and Metabolism, Food Functionality Research, Korea Food Research Institute, Jeollabuk-do, Republic of Korea

## Abstract

Bone homeostasis is dynamically balanced between bone forming osteoblasts and bone resorbing osteoclasts. Osteoclasts play an important role in bone destruction and osteoporosis, and they are derived from monocyte/macrophages in response to macrophage colony-stimulating factor (M-CSF) and receptor activator of nuclear factor *κ*B (NF-*κ*B) ligand (RANKL). *Amaranthus mangostanus* L. (AM) is a plant with powerful antioxidant and other biological activities including anti-inflammatory, antidiabetic, and antihyperlipidemic effects. However, its effects on bone health are unknown. In this study, we explored whether AM could affect RANK-mediated osteoclastogenesis. AM significantly suppressed RANKL-induced osteoclast differentiation and expression of osteoclast-specific genes, TRAP, cathepsin K, NF-activated T-cells (NFATc1), and Dc-stamp in RAW 264.7 cells. Moreover, AM significantly inhibited extracellular signal-regulated kinase (ERK), Akt, and NF-*κ*B signaling pathways in RAW 264.7 cells. In addition, AM preserved ovariectomy-induced bone loss in mice. Taken together, our results suggest that AM might be a potential candidate for the treatment of postmenopausal osteoporosis.

## 1. Introduction

Osteoporosis is a metabolic bone disease associated with low bone mass and microarchitectural failure of bone and increased risk of bone fractures. It develops mainly in postmenopausal women and increases their risk of bone fractures [1, 2].

Bone homeostasis is dynamically balanced between bone-forming osteoblasts and bone-resorbing osteoclasts [3]. Osteoclasts are large, multinucleated cells derived from monocyte/macrophage lineage cells in response to macrophage colony-stimulating factor (M-CSF) and receptor activator of nuclear factor *κ*B (NF-*κ*B) ligand (RANKL) [4, 5]. Binding of RANKL to RANK is a key factor for osteoclast differentiation and functions, and the resulting complex leads to the recruitment tumor necrosis factor (TNF) receptor-associated factors (TRAFs) and activates downstream signaling pathways, mainly NF-*κ*B, mitogen-activated protein kinase (MAPK), and AKT. Furthermore, it activates transcription factors such as nuclear factor of activated T cells (NFATc1) and c-fos to induce osteoclastogenesis [6–8].

Estrogen has an osteoprotective effect on bone remodeling, inhibits RANKL, and stimulates osteoblast to secrete osteoprotegerin (OPG), which competes with RANKL to suppress osteoclast differentiation [9, 10]. However, deficiency of estrogen after menopause results in increased osteoclastogenesis and decreased osteogenesis, which induces rapid bone loss and progression of osteoporosis [11]. Therefore, targeting RANKL-stimulated osteoclastogenesis may be a novel, alternative approach for postmenopausal osteoporosis treatment.

Considerable evidence shows that plant-derived natural products and traditional foods can promote bone mass and decrease bone fracture by suppressing osteoclastogenesis postmenopausally [12]. Edible Amaranth (*Amaranthus mangostanus* L., AM), known as “bireum” in Korea, is widely cultivated in Asia owing to its fast growth and nutritious value and belongs to the family Amaranthaceae [13, 14]. Amaranth is a plant with potent antioxidant capacity, and it also contains bioactive compounds such as *β*-carotene, polyphenol, anthocyanins, and lutein [15]. In addition, amaranth has recently been demonstrated to possess various biological activities such as anti-inflammatory, antidiabetic, and antihyperlipidemic activities in vivo [16–19]. However, the effects of AM on bone health have not been elucidated. Therefore, we sought to characterize the effects of AM on osteoclastogenesis and explore its underlying mechanisms in RAW 264.7 cells. Furthermore, we investigated the preventive and therapeutic effects of AM on an ovariectomy- (OVX-) induced bone loss in mice.

## 2. Materials and Methods

### 2.1. Reagents

RANKL was obtained from R&D Systems (Minneapolis, MN, USA). Dulbecco's modified Eagle's medium (DMEM), fetal bovine serum (FBS), and penicillin/streptomycin were purchased from Welgene (Daegu, Korea). Cell medium: minimum essential medium-*α* (*α*-MEM) was purchased from Gibco (USA). The tartrate-resistant acid phosphatase (TRAP) staining kit was obtained from Sigma-Aldrich. Primary antibodies against total and phospho-extracellular signal-regulated kinase (ERK), c-Jun N-terminal (JNK), p38, AKT, p-65, and NF-*κ*B inhibitor-*α* (I*κ*B*α*) were purchased from Cell Signaling Technology (Beverly, MA, USA). NFATc1 antibody was purchased from Santa Cruz Biotechnology (Santa Cruz, CA, USA).

### 2.2. Preparation of AM Extract

**Figure 1:**
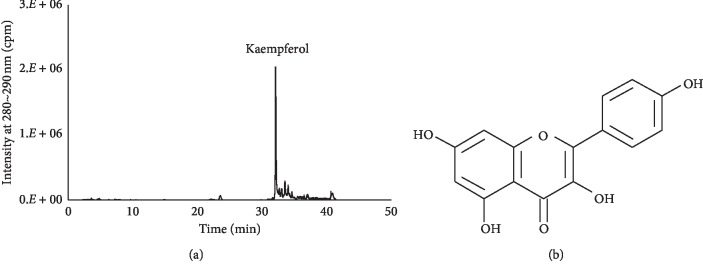
UPLC profile of *Amaranthus mangostanus* (AM) extract at 280∼290 nm (a). Chemical structure of kaempferol (b).

Dried AM was obtained from a local market in Korea, and the dried material was extracted (1 : 10 w/v) with 50% ethanol by shaking for 24 h at 20–25°C. The extract was collected using a filter paper (Whatman, USA) and vacuum evaporated. The supernatant was freeze-dried and stored at −80°C in a deep freezer. Major compounds present in the AM extract were analyzed using UPLC-MS (1290 Infinity II, USA; AB SCIEX, TripleTOF® 5600 plus, Agilent, USA) (Figure 1).

### 2.3. Analysis of AM Extracts Using LS-MS

To analyze and identify flavonols present in the AM extract, the UPLC-MS system was used. The UPLC-MS study (1290 Infinity II, USA; AB SCIEX, TripleTOF® 5600 plus, Agilent, USA) was conducted with a YMC-Triart C18 column (4.6 mm × 250 mm, 5 *μ*m, YMC Korea, Seongnam, Korea), and 0.1% formic acid in DW (mobile phase A) and 0.1% formic acid in acetonitrile (mobile phase B) were used as the mobile phases. The mobile phase B concentration was 25% at 0 min, 40% at 25 min, and 90% at 30 min. Electrospray ionization (ESI) was performed in the negative-ion mode. The desolvation temperature was 400°C, and the other temperatures were modified slightly from those described previously [20].

### 2.4. Cell Culture, Differentiation of Osteoclasts, and TRAP Activity

RAW 264.7 cells were purchased from the American Type Culture Collection (ATCC, Manassas, VA, USA) and cultured in DMEM supplemented with 10% FBS at 37°C. RAW 264.7 cells were plated in a 48-well plate at a density of 2 × 10^4^ cells/well. Osteoclast differentiation was induced by incubating the cells with M-CSF and RANKL in the presence or absence of various concentrations of AM. After 4 days, the cells were fixed and stained with TRAP solution, and then the TRAP-positive multinucleated cells containing three or more nuclei were considered osteoclasts and counted.

### 2.5. Cell Viability

The effect of AM on cell viability was evaluated using the 3-(4,5-dimethylthiazol-2-yl)-2,5-diphenyltetrazolium bromide (MTT) assay (Sigma). Cells were plated in a 96-well plate (1 × 10^4^ cells/well), and after 4 days of incubation, the cells were treated with varying concentrations of AM and then 0.5 mg/mL MTT solution was added to each well, followed by further incubation for 3 h at 37°C. The purple formazan crystals formed in the live cells were dissolved with dimethyl sulfoxide (DMSO), and the absorbance of the resultant solution was measured at 450 nm using an enzyme-linked immunosorbent assay (ELISA) reader (Molecular Devices, CA, USA).

### 2.6. RT-PCR Analysis

We evaluated the RANKL-induced expression of osteoclast-related genes using reverse transcription-polymerase chain reaction (RT-PCR). Briefly, total RNA was extracted using a QIAGEN mini kit (Qiagen, Valencia, CA, USA) according to the manufacturer's protocol. Real-time quantitative PCR (qPCR) was performed using the iTaq Universal SYBR Green I Supermix (Bio-Rad, Hercules, CA, USA) according to the supplier's protocol. The mRNA expression levels were normalized to those of glyceraldehyde 3-phosphate dehydrogenase (GAPDH) expression. The PCR primer sequences used were as follows: NEATc1, sense: 5ʹ-CCG TTG CTT CCA GAA AAT AAC A-3ʹ and antisense: 5ʹ-TGT GGG ATG TGA ACT CGG AA-3′; cathepsin K, sense: 5ʹ-CAG TAG CCA CGC TTC CTA TCC-3ʹ and antisense: 5ʹ-ACT GGG TGT CCA GCA TTT CC-3ʹ; TRAP, sense: 5ʹ-GCA GCC AAG GAG GAC TAC-3ʹ and antisense 5ʹ-CCC ACT CAG CAC ATA GCC-3ʹ; DC-stamp, sense: 5ʹ-TAT CTG CTG TAT CGG CTC A-3ʹ and antisense: 5ʹ-AGA ATA ATA CTG AGA GGA ACC CA-3ʹ; and GAPDH, sense: 5′-AAA TGG TGA AGC TCG CTC TG-3′ and antisense: 5′-TGA AGG GGT CGT TGA TGG-3′.

### 2.7. Western Blot Analysis

To further corroborate the effects of the extract on osteoclast-related genes, protein levels were examined using Western blot analysis. Briefly, the whole cells were washed with PBS and lysed in ice-cold radioimmunoprecipitation assay (RIPA) buffer (40 mM HEPES (pH 7.4), 120 mM sodium chloride (NaCl), 1 mM ethylenediaminetetraacetic acid (EDTA), 50 mM sodium fluoride (NaF), 1.5 mM sodium vanadate (Na_3_VO_4_), 10 mM *β*-glycerophosphate, and 1% Triton X-100) supplemented with a protease and phosphatase inhibitor cocktail. Cell lysates were centrifuged at 13,000 ×g at 4°C for 15 min. To confirm the translocation of p65, nuclear and cytoplasmic proteins were extracted using nuclear extract kit (Active Motif Co., Carlsbad, CA, USA), according to the manufacturer's instructions. Equal amounts of protein were separated using sodium dodecyl sulfate-polyacrylamide gel electrophoresis (SDS-PAGE) and transferred onto nitrocellulose (NC) membranes. The membranes were blocked with 5% skim milk for 1 h and incubated overnight with primary antibodies at 4°C and then with horseradish peroxidase-conjugated secondary anti-mouse or anti-rabbit IgG (1 : 1,000, Santa Cruz Biotechnology Inc.) for 1 h. Signals were detected using a chemiluminescence reagent (Amersham Bioscience, Piscataway, NJ, USA).

### 2.8. Animals and Ovariectomy

This animal study protocol was approved by the Institutional Animal Care and Use Committee of the Korea Food Research Institute (KFRI: KFRI-M-17047). Thirty ICR female mice (8 weeks old) were obtained from Orient-Bio Korea (Seoul, Korea) and were housed under controlled conditions at a temperature of 22–26°C on a 12 h light/dark cycle. All the mice were divided into three groups, and two group were subjected to ovariectomies, whereas one group underwent bilateral surgery (SHAM, *n* = 10). Two weeks later, the 20 OVX rats were body-weight matched and randomly divided into the vehicle-treated OVX (OVX) and AM-treated OVX (AM, 5 g/kg in the diet) groups. AM was suspended in distilled water while the diets were prepared by mixing each of the above-specified components with 1 kg of AIN 93M powdered diet (Dyets Inc., Bethlehem, PA, USA). Mice in the SHAM and OVX groups were fed a soy-free control diet and their body weight was measured weekly. The mice were subsequently euthanized and blood samples were collected via heart puncture and stored at −70°C for biochemical analysis. The femurs were excised and fixed in 4% paraformaldehyde at 4°C for bone structural parameter determination.

### 2.9. Serum Biochemical Assay

Serum total cholesterol (TC), high-density lipoprotein cholesterol (HDL-C), and triglyceride (TG) levels were determined using commercial enzyme kits (Asan Pharmaceuticals, Hwasung, Korea). Low-density lipoprotein cholesterol (LDL-C) levels were calculated as follows: LD L-C = TC – HDL-C – TG/5.

### 2.10. Microcomputed Tomography (*μ*CT) Scanning

The fixed femurs were analyzed using a microcomputed tomography (*μ*CT) scanner system (SkyScan 1076, Bruker, Germany). The scanning procedure was set at 18 *μ*m and exposures were performed at 100 kV, 100 mA, and 1770 ms/frame. The CT images were scanned and analyzed using the relevant software (CT Analyzer V 1.11.0.0, Skyscan, Kontich, Belgium), and the two-dimensional (2D) and 3D images were obtained for visualization and analysis. The following bone morphometric parameters were evaluated, bone volume to tissue volume ratio (BV/TV), trabecular number (Tb.N), trabecular thickness (Tb.Th), and trabecular separation (Tb.Sp), using images acquired from a CT-AN 1.13 instrument (Bruker, Germany). The scan analysis operator was blinded to the specific treatments associated with each specimen.

### 2.11. Statistical Analysis

In vitro and in vivo data are presented as means ± standard error of the mean (SEM). The results were analyzed using a one-way analysis of variance (ANOVA) followed by Tukey's post hoc test using the GraphPad Prism software version 6.05 (Graphpad Software Inc., La Jolla, CA, USA). Differences between groups were considered significant at *p* < 0.05.

## 3. Results

### 3.1. AM Is Not Cytotoxic to RAW 264.7 Cells

**Figure 2:**
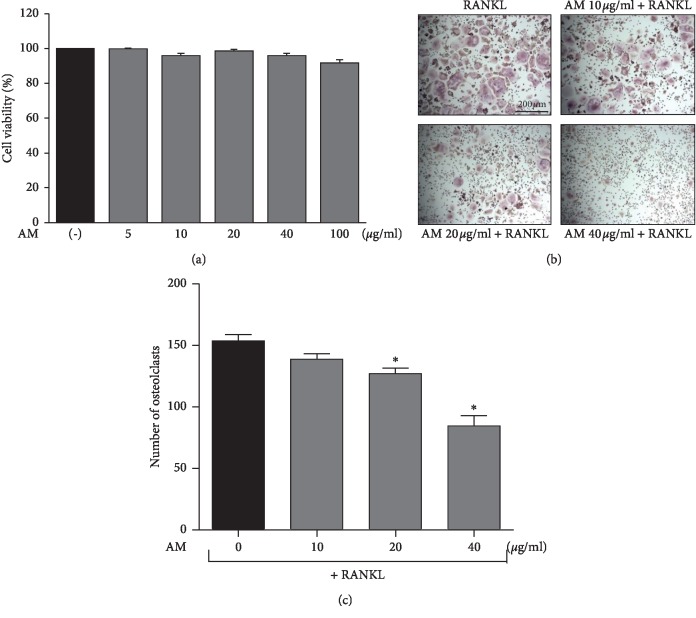
*Amaranthus mangostanus* (AM) inhibited receptor activator of nuclear factor-*κ*B (NF-*κ*B) ligand- (RANKL-) induced osteoclastogenesis without cytotoxicity in vitro. (a) Effects of AM on cell viability. RAW 264.7 cells were cotreated with RANKL (50 ng/mL) and AM (0–100 *μ*g/mL) for 4 days. (b) RAW 264.7 cells were treated with indicated concentrations of AM followed by RANKL (50 ng/mL) stimulation for 4 days. (c) Tartrate-resistant acid phosphatase- (TRAP-) positive cells with more than three nuclei were considered osteoclast; *n* = 3; ^*∗*^*p* < 0.05 compared with control group.

To evaluate the effects of AM on cell viability, we determined its cytotoxicity against RAW 264.7 cells and found that it had no significant effect on the viability of these cells (Figure 2(a)).

### 3.2. AM Inhibits RANKL-Induced Osteoclastogenesis in RAW 264.7 Cells

To determine the effects of AM on osteoclastogenesis, RAW 264.7 cells were incubated with RANKL in presence or absence of various concentrations of AM. RANKL-treated cells differentiated into TRAP-positive multinucleated osteoclasts, and AM treatment decreased this effect in a dose-dependent manner (Figures 2(b) and 2(c)).

### 3.3. AM Suppresses RANKL-Induced Osteoclast-Mediated Genes Expression

**Figure 3:**
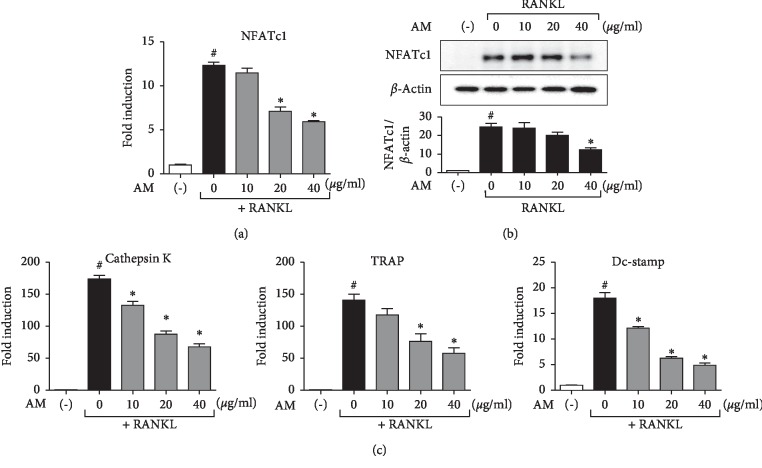
*Amaranthus mangostanus* (AM) suppressed receptor activator of nuclear factor-*κ*B (NF-*κ*B) ligand- (RANKL-) induced expression of osteoclast-specific genes. (a)–(d) RAW 264.7 cells were cultured with RANKL (50 ng/mL) at various concentrations for 4 days, and osteoclast-specific genes (cathepsin K, TRAP, Dc-stamp, and nuclear factor of activated T-cells (NFATc1)) were analyzed using real-time polymerase chain reaction (PCR). RNA expression levels were normalized to expression levels of glyceraldehyde 3-phosphate dehydrogenase (GAPDH). (e) RAW 264.7 cells were cultured with RANKL (50 ng/mL) with various concentrations for 4 days and osteoclast-specific gene NFATc1 was analyzed using Western blotting; *n* = 3; ^#^*p* < 0.05 compared with control group, and ^*∗*^*p* < 0.05 compared with RANKL treatment group.

RANKL has been shown to induce the expression of osteoclast differentiation-related genes including NFATc1, TRAP, cathepsin K, and Dc-stamp. Therefore, to investigate the effects of AM on osteoclastogenesis, we evaluated RANKL-induced mRNA expression of these osteoclast-related genes using RT-qPCR. The results showed that AM decreased the expression levels of these genes in a dose-dependent manner (Figures 3(a) and 3(c)). To further confirm the effect of AM on the mRNA expression of NFATc, we also measured its protein level using immunoblot analysis. AM treatment inhibited RANKL-induced NFATc1 protein levels in a dose-dependent manner (Figure 3(b)).

### 3.4. AM Inhibits RANKL-Induced Activation of MAPKs, Akt, and NF-*κ*B Pathways

**Figure 4:**
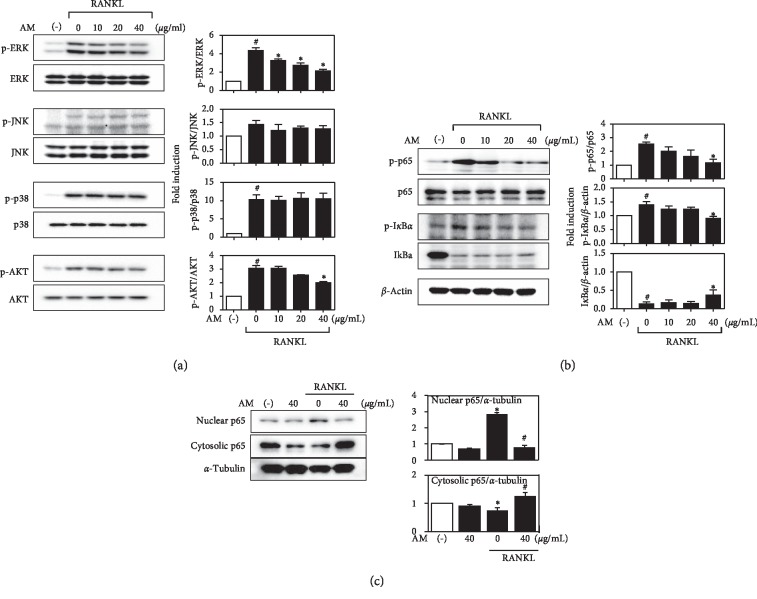
*Amaranthus mangostanus* (AM) inhibits osteoclast differentiation via nuclear factor-*κ*B (NF-*κ*B) ligand- (RANKL-) induced mitogen-activated protein kinase (MAPK) signaling pathways. (a)–(c) RAW 264.7 cells were treated with AM at indicated concentrations, followed by RANKL (50 ng/mL). (a) Whole cell lysates were analyzed using Western blot using specific antibodies against phospho-extracellular signal-regulated kinase (ERK), ERK, phospho-p38, p38, phospho-c Jun N-terminal (JNK), JNK, phospho-Akt, and Akt and (b) phospho-p65, p65, phospho-I*κ*B, I*κ*B, and *β*-actin. (c) The nuclear and cytosolic p65 were analyzed using Western blot. Relative ratios of phosphorylation bands to total bands density were quantified using National Institutes for Health (NIH) ImageJ software and values are indicated under each band; *n* = 3; ^#^*p* < 0.05 compared with control group, and ^*∗*^*p* < 0.05 compared with RANKL treatment group.

RANKL-stimulated osteoclastogenesis is mediated by signaling pathways such as MAPKs, Akt, and NF-*κ*B [21]. To investigate the effect of AM on RANKL-stimulated signaling pathways mediating osteoclast differentiation in RAW 264.7 cells, we determined the phosphorylation of MAPKs (ERK, JNK, and p38), Akt, and NF-kB using immunoblot analysis. Treatment of RAW 264.7 cells with RANKL dramatically phosphorylated ERK, JNK, p38 MAPKs, and Akt, as well as NF-*κ*B signaling pathway (p-65 and I*κ*B*α* phosphorylation and I*κ*B*α* degradation) molecules. AM significantly inhibited RANKL-induced ERK and Akt activation in a dose-dependent manner. However, phosphorylated p38 and JNK MAPKs did not significantly change (Figure 4(a)). AM also largely suppressed the RANKL-induced NF-*κ*B signaling pathway (Figure 4(b)). Moreover, AM attenuated RANKL-induced translocation of p65 from the cytosol to the nucleus (Figure 4(c)).

### 3.5. AM Inhibits the Body, Fat, Liver, and Uterine Weight in OVX-Induced Mice

AM was further evaluated for its effects on osteoporosis. All three groups of rats showed similar initial body weights, whereas the final body weight of the OVX group was dramatically higher than that of the SHAM group. However, the mean final body weight of the AM-treated group was significantly reduced, suggesting that AM inhibited the OVX-induced body weight gain. Furthermore, the fat and liver weight of the OVX group was considerably greater than that of the SHAM group; however, that of the AM group was significantly reduced. Furthermore, the uterine weight of the OVX group was reduced more than that of the SHAM group, but no change was observed in the uterine weight of the AM group (Table 1).

### 3.6. AM Inhibits the Lipid Metabolism in OVX-Induced Mice

**Figure 5:**
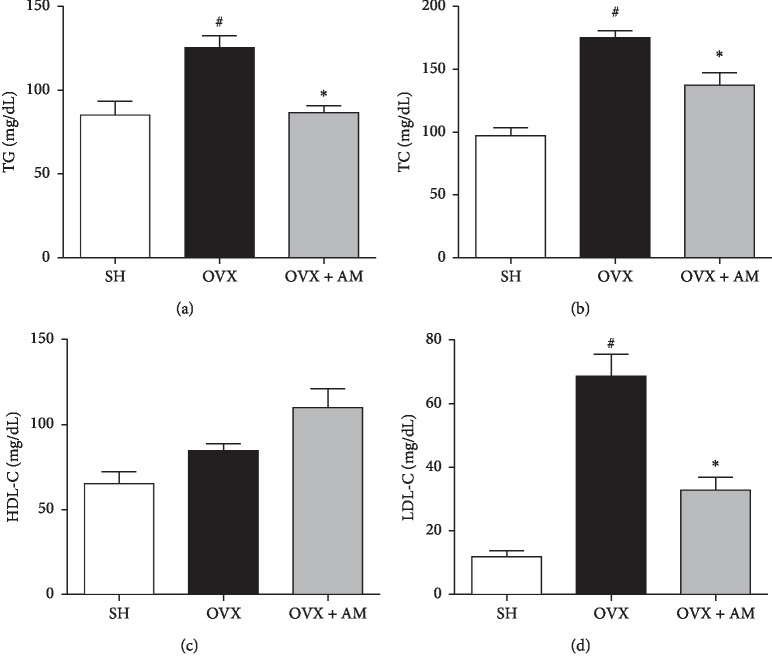
*Amaranthus mangostanus* (AM) improves ovariectomy-induced dyslipidemia in mice. (a) Triglycerides (TG) and (b) total cholesterol (TC). (c) High-density lipoprotein cholesterol (HDL-C). (d) Low-density lipoprotein cholesterol (LDL-C). ^#^*p* < 0.05 and ^*∗*^*p* < 0.05 compared to SHAM and OVX groups, respectively.

TG, TC, and LDL levels were significantly higher in the OVX mice than they were in the SHAM mice, whereas the levels were lower in the AM group than they were in the OVX mice. In addition, the group supplemented with AM exhibited slightly higher HDL levels than those of the OVX group (Figure 5).

### 3.7. AM Inhibits the Bone Loss in OVX-Induced Group In Vivo

**Figure 6:**
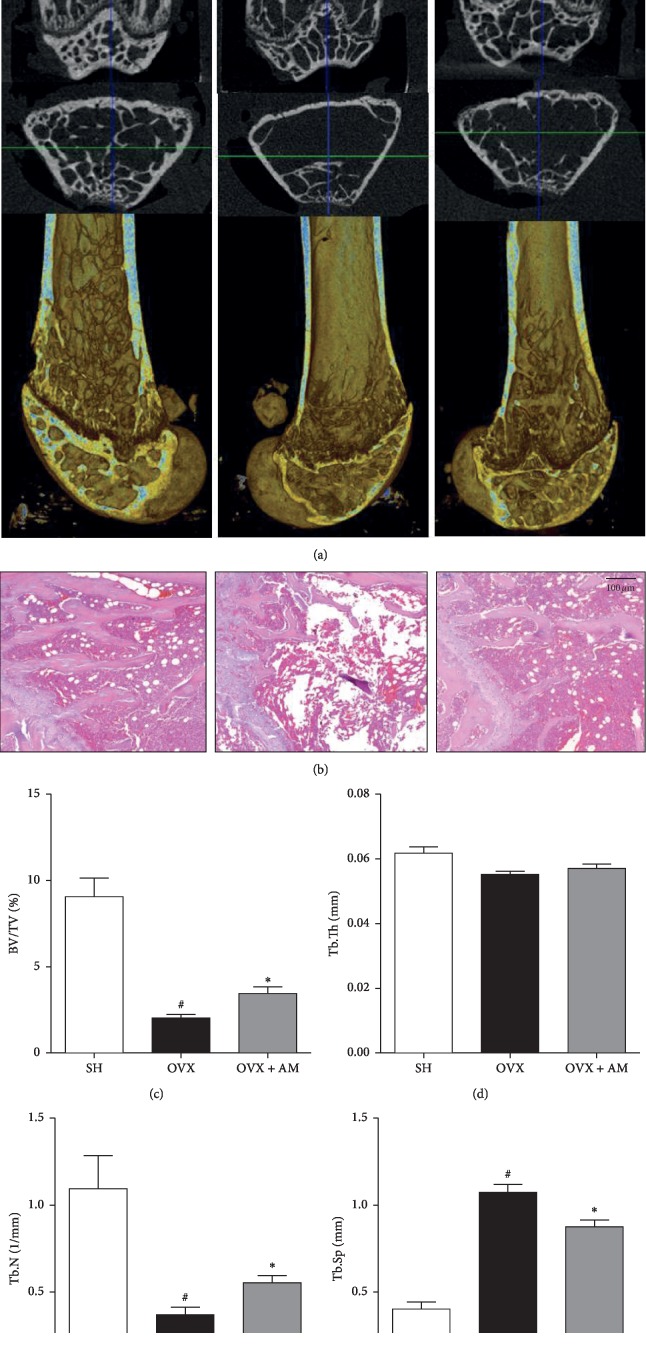
*Amaranthus mangostanus* (AM) prevents ovariectomy-induced bone loss in mice. (a) Representative two-dimensional (2D) and 3D images of trabecular bone in femurs analyzed using microcomputed tomography (*μ*CT). (b) Hematoxylin and eosin (H&E) staining, scale bar indicates 100 *μ*m. (c) Bone volume to tissue volume ratio (BV/TV) of femur. Bone parameters of the femur: (d) trabecular thickness (Tb.Th), (e) trabecular number (Tb.N), and (f) trabecular separation (Tb.Sp). Data are mean ± standard error of the mean (SEM); ^#^*p* < 0.05 and ^*∗*^*p* < 0.05 compared to SHAM and OVX groups, respectively.

To evaluate the effect of AM on OVX-induced bone deterioration, we examined the trabecular microarchitecture using *μ*CT and hematoxylin and eosin (H&E) staining of the distal femur of the mice. First, the 3D images revealed differences in the trabecular microarchitecture in the various groups. There was considerable deterioration of the trabecular bone of the OVX mice, whereas the AM-treated mice exhibited significant inhibition of OVX-induced deterioration of the bone structure (Figure 6(a)). The bone-morphometric parameters examined were the BV/TV, Tb.N, Tb.Th, and Tb.Sp. Quantification of the trabecular bone response found that the OVX group had decreased BV/TV and Tb.N and increased Tb.Sp compared with values of the SHAM group. Furthermore, AM increased the BV/TV and Tb.N and reduced the Tb.Sp compared to those of the OVX mice (Figures 6(c)–6(f)). However, the level of the Tb.Th was not different among the groups. Furthermore, the H&E staining shows that the AM groups exhibited a more significant preservation of the microtrabecular bone than the OVX groups did (Figure 6(b)).

## 4. Discussion

Osteoporosis is a “silent” disease, which commonly causes no symptoms until a fracture occurs [22]. Osteoporotic fractures are one of the most dangerous occurrences in patients and can cause further disability and early mortality. Moreover, it leads to huge economic cost to society. Bone homeostasis is a dynamic process regulated by bone formation and resorption mediated by osteoblastic and osteoclastic activities. However, an imbalance in these processes such as excessive bone resorption caused by enhanced osteoclastogenesis could cause bone-associated diseases including osteoporosis and bone metastasis [23]. Therefore, targeting the downregulation of osteoclastic signals as an alternative treatment strategy for osteoporosis is useful.

Osteoclasts are large, multinucleated cells involved in bone resorption, and they are derived when hematopoietic progenitor differentiates mononuclear TRAP-positive preosteoclast cells and fuses the multinucleated mature osteoclasts [23]. AM inhibited TRAP activity and the formation of TRAP-positive multinucleated osteoclasts and did not cause any cytotoxicity in RAW 264.7 cells (Figure 2). These findings suggest that AM inhibited osteoclastogenesis.

RANKL has a crucial role in osteoclastogenesis, and its binding to RANK stimulates TNF receptor-associated factor 6 (TRAF6), which activates downstream signaling pathways such as MAPKs, Akt, and NF-*κ*B involved in osteoclast differentiation [24–26]. MAPKs including ERK, JNK, and p38 pathways phosphorylated and the generated signals are transduced to the nucleus for gene transcription. Phosphorylation of ERK reduces osteoclast differentiation. Inhibition of the ERK pathway decreases osteoclast formation [27]. The present results showed that AM suppressed phosphorylation of ERK and the activation of the NF-*κ*B pathway was associated with five member proteins such as RelA (p65), RelB, cRel, NF-*κ*B1 (p50), and NF-*κ*B (p52).

One study reported that NF-*κ*B p50/p52 knockout mice exhibited severe osteoporosis because osteoclasts did not form [24]. In resting cells, NF-*κ*B proteins are in the cytoplasm combined with the inhibitory I*κ*B*α* [28]. Following the binding of RANKL to RANK, I*κ*B*α* is phosphorylated and degraded, and p65/p50 dimers are translocated to the nucleus for gene transcription [25]. These results reveal that AM reduced RANKL-induced phosphorylation of p65 and blocked the degradation of I*κ*B. In addition, RANKL activates the Akt signal pathway by interacting with TRAF6 and c-Src. These results show that AM reduced RANKL-induced phosphorylation of Akt (Figure 4), suggesting that AM suppressed osteoclastogenesis by suppressing RANKL-induced ERK, NF-*κ*B, and Akt signaling pathways.

NFATc1 is considered a key transcription factor of NF-*κ*B, ERK, and Akt signaling pathways in osteoclastogenesis [29]. This transcription factor is essential in regulating osteoclast-specific genes such as TRAP, cathepsin K, and Dc-stamp [30]. Our results showed that AM significantly inhibited the expression of NFATc1 and the osteoclastic genes, TRAP, cathepsin K, and Dc-stamp (Figure 3). These results suggest that AM affected the NFATc1 and downstream gene expression.

OVX mice are a well-known experimental model of postmenopausal osteoporosis associated with estrogen deficiency and exhibit a similar reduction in the BMD and change of microarchitecture to that shown in humans [31]. Furthermore, excessive activation of osteoclastogenesis is a key factor in the pathogenesis of osteoporosis [32]. Thus, inhibiting BMD and bone fracture by suppressing osteoclastogenesis may be a useful strategy for postmenopausal osteoporosis treatment. Several bodies of evidence demonstrated that preservation of trabecular bone microarchitecture improved, reducing the fracture risk and bone strength [33].

Our study showed that AM suppresses osteoclastogenesis in vitro (Figure 2) and administration of AM significantly reserved the OVX-induced trabecular bone parameters in vivo (Figure 6). These results suggest that AM prevented bone mass and trabecular bone by suppressing osteoclastogenesis. In addition, postmenopausal women with deficiency of estrogens show obesity, hyperlipidemia, and insulin resistance [34]. In our previous study, we reported that OVX mice exhibited increased body weight, hyperlipidemia, and visceral fat accumulation [35]. In the present study, the OVX mice showed increased body, fat, and liver weight compared to the SHAM mice. Treatment with AM protected mice against the OVX-induced increase in body, liver, and fat weight (Table 1). Furthermore, AM also reduced the serum LDL-C and TG in OVX mice (Figure 5). These results suggest that AM prevented obesity-mediated metabolic diseases in OVX mice.

Recently, it was shown that an extract of AM containing *β*-carotene, polyphenol, anthocyanin, and lutein has various biological activities such as antioxidant and anti-inflammatory effects [15]. In this study, we evaluated one of the compounds of AM extract, kaempferol. Several studies have shown that kaempferol inhibits osteoclastic bone resorption and stimulates osteogenic differentiation in vitro [36, 37]. Moreover, kaempferol attenuated bone loss in a rat model of OVX-induced osteopenia [38]. Therefore, it was considered that kaempferol present in the AM extract is one of the bioactive compounds responsible for the antiosteoporotic effects.

In the present study, we, for the first time, provide evidence that AM effectively suppressed RANKL-induced osteoclast differentiation by inhibiting the NF-*κ*B and MAPK signaling pathways in vitro. In addition, AM prevented OVX-induced bone loss in vivo. Thus, our finding results suggest that AM is a promising alternative treatment candidate for osteoporosis.

## Figures and Tables

**Table 1:** Effects of *Amaranthus mangostanus* (AM) on body, fat, liver, and uterine weights of ovariectomized (OVX) mice.

	SHAM	OVX	OVX + AM
Initial body weight (g)	28.15 ± 0.35	29.05 ± 0.14	28.83 ± 0.47
Final body weight (g)	41.55 ± 1.54	57.29 ± 1.06^#^	51.09 ± 1.23^*∗*^
Fat weight (g)	0.366 ± 0.026	0.716 ± 0.034^#^	0.541 ± 0.038^*∗*^
Liver weight (g)	1.718 ± 0.068	2.341 ± 0.073^#^	1.997 ± 0.046^*∗*^
Uterine weight (g)	0.268 ± 0.042	0.050 ± 0.003^#^	0.048 ± 0.006

^#^ *p* < 0.05 and ^*∗*^*p* < 0.05 compared to SHAM and OVX groups, respectively.

## Data Availability

The data used to support the findings of this study are available from the corresponding author upon request.
